# An Efficient and Uncertainty-Aware Decision Support System for Disaster Response Using Aerial Imagery

**DOI:** 10.3390/s22197167

**Published:** 2022-09-21

**Authors:** Junchi Bin, Ran Zhang, Rui Wang, Yue Cao, Yufeng Zheng, Erik Blasch, Zheng Liu

**Affiliations:** 1 School of Engineering, Okanagan Campus, University of British Columbia, Kelowna, BC V1V 1V7, Canada; 2 Department of Data Science, University of Mississippi Medical Center, Jackson, MS 39216, USA; 3 MOVEJ Analytics, Dayton, OH 45324, USA

**Keywords:** aerial imagery, building damage assessment, information fusion, robust operation, model efficiency

## Abstract

Efficient and robust search and rescue actions are always required when natural or technical disasters occur. Empowered by remote sensing techniques, building damage assessment can be achieved by fusing aerial images of pre- and post-disaster environments through computational models. Existing methods pay over-attention to assessment accuracy without considering model efficiency and uncertainty quantification in such a life-critical application. Thus, this article proposes an efficient and uncertain-aware decision support system (EUDSS) that evolves the recent computational models into an efficient decision support system, realizing the uncertainty during building damage assessment (BDA). Specifically, a new efficient and uncertain-aware BDA integrates the recent advances in computational models such as Fourier attention and Monte Carlo Dropout for uncertainty quantification efficiently. Meanwhile, a robust operation (RO) procedure is designed to invite experts for manual reviews if the uncertainty is high due to external factors such as cloud clutter and poor illumination. This procedure can prevent rescue teams from missing damaged houses during operations. The effectiveness of the proposed system is demonstrated on a public dataset from both quantitative and qualitative perspectives. The solution won the first place award in International Overhead Imagery Hackathon.

## 1. Introduction

### 1.1. Application Background

**Figure 1:**
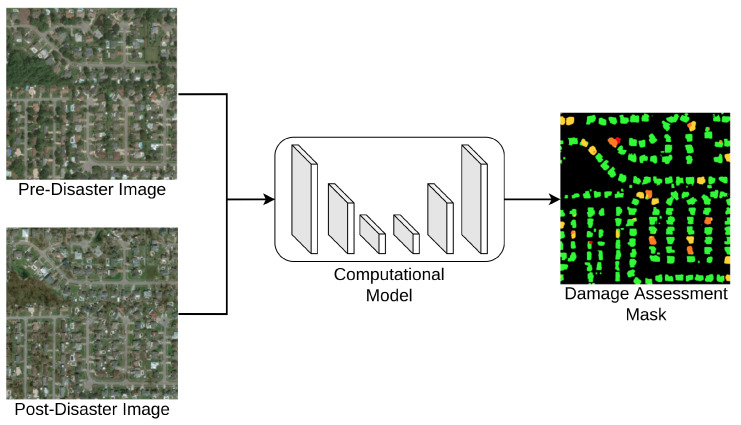
The illustration of the computational model for building damage assessment.

In the past few decades, global climate change has significantly increased the frequency of natural disasters such as earthquakes, floods, and tsunamis [1]. These disasters have caused not only severe economic loss, but also numerous casualties. After the natural disasters, many brave government staves and volunteers devote themselves to disaster response for search and rescue human life from ruins. Before the rescue operations begin, it is essential to localize and locate buildings and assess the damage levels of houses to create an efficient plan to save as many people as possible. Such a screening procedure is called building damage assessment (BDA). With advanced sensory technologies, modern satellites are equipped with optical sensors to provide high-resolution aerial images over time. When disasters occur, the search and rescue teams rely on the recorded aerial images to estimate the damage levels of houses in order to plan rescue actions. The damage levels are usually categorized as “No Damage”, “Minor Damage”, “Major Damage”, or “Destroyed”. According to the severity of the damaged buildings, the rescue team determines the priority of buildings for final search and rescue operations. Although aerial images enable rescue teams to plan the operations effectively, it is still challenging to manually assess all the buildings in a timely manner. An automatic system is desired to quickly screen and assess the buildings to support the rescue team, especially for such emergent scenarios. In recent years, emerging data-driven computational models such as deep learning models [2] have drawn much attention due to their powerful representation learning in computer vision tasks. With sufficient annotated datasets, the deep learning models can learn the features for predictive tasks such as object detection [3], semantic segmentation [4], etc. Thus, several researchers [1,5,6] also introduce deep learning as a computational model for BDA. The general architecture of these solutions can be summarized as shown in Figure 1. The key idea aims to estimate damage to the houses through imagery comparison between the pre- and post-disaster, assisted by a computational model, as shown in Figure 1. The pre-disaster images visualize the shapes and texture of intact houses, while the post-disaster images show the corresponding information impacted by natural or technical disasters. Then, the computational models compare the two kinds of images to generate the damage assessment masks.

### 1.2. Related Work

According to the nature of the computational models in BDA, it can be categorized into two groups, i.e., an end-to-end model and ensemble models. Then, end-to-end models aim to address the challenge based on the recent advances in deep learning. For example, Weber et al. [5] incorporate shared-weight residual networks (ResNet) [7] for feature extraction. Then a feature pyramid network (FPN) [8] is used to decode the final building damage maps via fusing features of pre- and post-disaster images. The feature fusion is through an addition operation. Gupta et al. [6] further introduces the DeepLabv3+ [9] to the applications of BDA with a modified dilated ResNet [7]. Moreover, Shen et al. [1] proposed a cross-fusion direction model, i.e., BDANet, to generate more precise maps of damage levels. Unlike the frameworks of Gupta et al. [6] and Weber et al. [5], the BDANet introduces visual attention to draw the correlation between pre- and post-disaster images to fuse the information instead of simple addition of concatenation. Specifically, the BDANet draws both spatial attention and channel attention through convolution neural networks (CNN) [1]. The introduction of vision attention significantly improves accuracy. However, the proposed BDANet still has room to improve by introducing more state-of-the-art vision attention methods, such as multi-head attention (MHA) in vision transformer (ViT) [10]. Nowadays, ViT [10] is the dominant fundamental model in computer vision after ResNet [7]. However, despite its powerful capability, the MHA features high complexity that prevents deployment from efficiency-critical scenarios. Lee-Thorp et al. [11] introduce the use of Fourier transform to approximate the MHA in natural language processing efficiently. However, there is still no implementation of computer vision tasks. Another significant aspect is the ensemble models. The U.S Defense Innovation Unit hosted a well-known international challenge, i.e., Xview2, to invite developers to facilitate the BDA based on computational models with a published dataset and a baseline model [12]. Around 500 teams participated in the challenge, with over 2000 submitted solutions. To further achieve higher accuracy in damage assessment, many participants ensemble numerous different trained models for this purpose [13]. The top solution [12,14] combines 12 different computational models, which achieve superior performance in this challenge. However, such ensemble methods are too inefficient to apply in real-world scenarios.

Beyond the limitation in efficiency, recent BDA methods lack the capability to quantify the uncertainty of the segmented objects, which may greatly increase the chance of missing potential targets. For such a safety-critical application, uncertainty quantification is essential, but there is no investigation yet in BDA. However, uncertainty quantification is rapidly growing in other applications, such as medical imaging [15,16] and autonomous driving [17,18]. Within these methods, an approximation of statistical sampling is required to output the mean and deviation of the probabilities of the segmentation. The deviation can reflect the predictive uncertainties [15]. There are two categories of approaches for the purpose, i.e., Monte Carlo Dropout (MC Dropout) [19] and ensemble modeling [20]. The MC Dropout aims to approximate the sampling by implementing dropout in the same neural network multiple times, while ensemble models collect numerous results from multiple independent networks [15,21]. Compared with the ensemble model, MC Dropout is much more efficient without training multiple networks, according to the empirical reports [15,21,22]. As mentioned, efficiency is one of the major concerns in recent BDA. In this regard, MC dropout is more suitable for the BDA while there is still a lack of investigation in this application.

### 1.3. Problem Statement

Despite the great success of these solutions [1,5,6,14] in BDA accuracy, the existing approaches are still limited in real-world applications of BDA. Specifically, the concerns are listed as follows:

- **Assessment Efficiency for Deployment**. Disaster responses are always demanded to take action as soon as possible in order to save victims. However, recent methods pay over-attention to assessment accuracy without concerning the inference time consumption, which makes them difficult to deploy.
- **Uncertainty Quantification for Decision Support**. It is noticeable that computational models are still an emerging topic without perfect performance in disaster response. The predictive failure may cause more casualties in a fully automatic manner without experts’ intervention. Thus, the BDA should also deliver the uncertainty of assessments as supplementary information other than assertive damage assessment masks for final decisions from experts.

### 1.4. System Overview and Contribution

**Figure 2:**
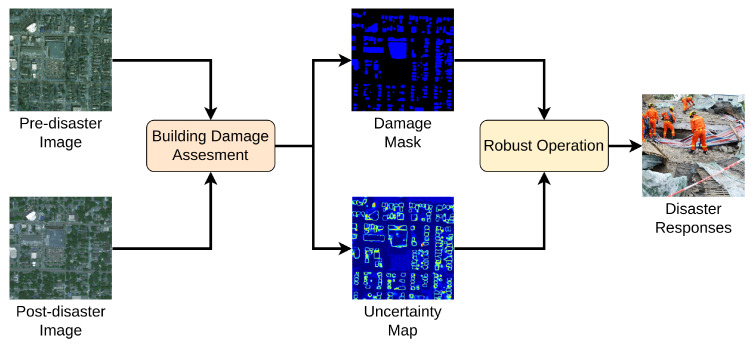
The illustration of the proposed efficient and uncertainty-aware decision support system (EUDSS).

In this regard, this article proposed a new approach, i.e., efficient and uncertain-aware decision support system (EUDSS), to support planning disaster responses as shown in Figure 2. Specifically, the EUDSS features two steps for this purpose, i.e., building damage assessment (BDA) and robust operation (RO). The BDA stage aims to fuse the pre-disaster and post-disaster images to automatically evaluate the damage levels of houses at the pixel level, which ranges from “No Damage” to “Destroyed”. In the BDA stage, a new method, i.e., efficient and uncertain-aware BDA (EUBDA), is proposed to generate the damage mask of buildings efficiently. Differing from contemporary methods, the EUBDA includes a proposed new attention method, namely Fourier attention (FA), to fuse pre- and post-disaster images efficiently. Meanwhile, the EUBDA introduces Monte-Carlo Dropout (MC Dropout) to quantify the uncertainties of the damage assessment simultaneously. Then, the derived damage mask and uncertainty map are fed and delivered into the RO for post-analysis. The RO aims to determine if an additional manual or expert review is required. For example, the damaged mask may indicate that there are no damaged houses in the monitoring regions while the uncertainty is high. Such a situation reflects the potential failed assessment at the BDA stage, which may cause missing victims to be buried under the ruins. Under the situation, the RO will invite the experts to review the assessment through a website interface. The revisited assessment will be used for the final decision on disaster responses. The contributions of the article are summarized as follows:

- In this study, an automatic two-stage decision system, i.e., EUDSS, is proposed to enable building damage assessment (BDA) to efficiently support decision-making during disaster response.
- In the first BDA stage, a computational model is proposed, i.e., efficient and uncertainty-aware BDA (EUBDA). The EUBDA first includes an innovative FA module to fuse pre- and post-disaster information. Then, the EUBDA employs MC Dropout to estimate the uncertainty maps with the damage assessment results. Both modules are new to the application domain of disaster response.
- The RO stage introduces a web application to support experts’ decisions regarding rescue plans with additional uncertainty maps. The case studies demonstrate the feasibility of RO in real-world scenarios (https://engineering.ok.ubc.ca/tag/overhead-imagery-hackathon/, accessed on 7 September 2022).

In summary, the proposed EUDSS evolves the recent BDA frameworks to a efficient and robust decision support system for disaster responses. The rest of the paper is organized as follows. Section 2 presents the proposed system in detail. Section 3 illustrates the experimental results. Finally, Section 4 concludes this article.

## 2. Efficient and Uncertainty-Aware Decision Support System

### 2.1. Building Damage Assessment for Initial Evaluation

**Figure 3:**
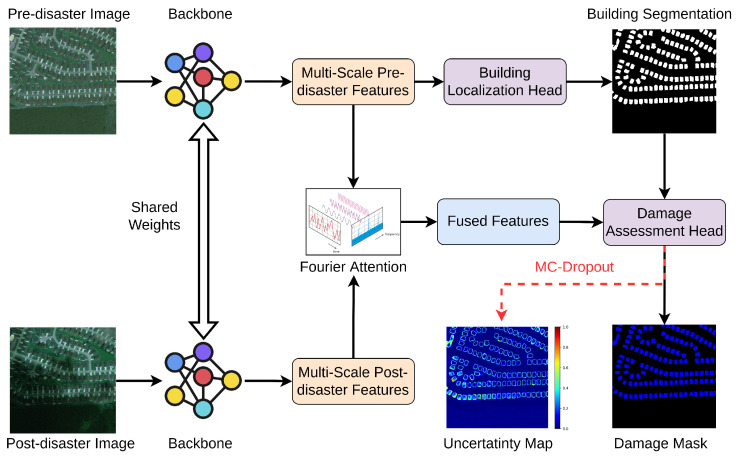
The illustration of efficient and uncertainty-aware building damage assessment (EUBDA).

Figure 3 visualizes the design of the proposed EUBDA for the initial evaluation of house damages. First, a primary neural network or backbone is implemented to extract the multi-scale features from both pre-disaster and post-disaster images. Meanwhile, the “shared weights“ indicate that the implemented backbones are the equivalent for these two images. The motivation of the design aims to extract aligned image features for further information fusion. Such a design can greatly reduce the memory occupation [1]. Second, the multi-scale pre-disaster features are fed into the building localization head to generate initial building segmentation, a binary mask. Simultaneously, the extracted multi-scale features from pre- and post-disaster images are fused by the proposed vision attention method, i.e., Fourier attention (FA). The FA efficiently fuses the information between the pre-disaster and post-disaster features in the frequency domain. Then, fused features multiply the building segmentation to make features concentrate where the buildings are. Finally, the damage assessment head estimates the damage levels resulting in a damage mask. Moreover, the head adopts Monte Carlo dropout (MC dropout) [19] to simulate the statistical sampling process for uncertainty quantification.

#### 2.1.1. Backbones for Multi-Scale Feature Extraction

As mentioned, the implemented backbone aims to extract the features from pre-disaster and post-disaster images in an efficient manner. In this research, the recent VovNet [23] models are adopted in this framework. Compared with standard ResNet [7], the VovNet also consists of five stages denoted as $F=\{F_{1},...,F_{5}\}$ after serial CNN blocks. Additionally, the VovNet applies channel attention [24] on extracted features across all stages that refine and enhance the features. It is noticeable that the first stage of the backbone is designed to normalize and project the raw data to latent space. In this regard, the extracted features are not feasible for complex operations such as information fusion. Thus, only $F=\{F_{2},F_{3},F_{4},F_{5}\}$ are included for further operation. The learned weights of the VovNet are reused on both pre- and post-disaster images for aligned features which are denoted as $F_{\mathrm{pre}}$ and $F_{\mathrm{post}}$ respectively.

#### 2.1.2. Fourier Attention for Feature Fusion

**Figure 4:**
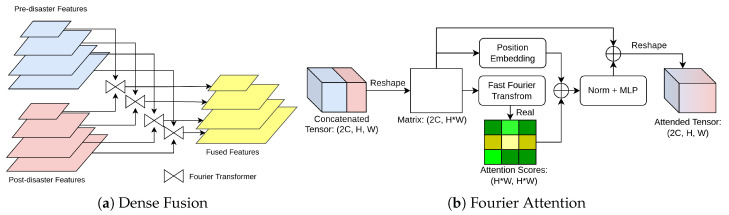
The illustration of a structure of dense fusion and the proposed Fourier attention (FA).

Inspired by the novel multimodal fusion method of Prakash et al. [25], it is recommended to develop ViTs as fusion operators to densely fuse the features of each stage in order to greedily integrate global context from each modality, as shown in Figure 4a. Nonetheless, ViT [10] is too large to directly train due to large matrix multiplication, even on the workstation with 16 GB of graphical memory. Meanwhile, the Fourier transform also offers a distinct view of features in the frequency domain. In this regard, the proposed Fourier attention (FA) replaces the multi-head attention module with 2D fast Fourier transform (FFT), which has comparable effectiveness to the original multi-head attention (MHA) with light memory footprint [11], as shown in Figure 4b. Specifically, the FA is formulated for each level of features from the backbone as:

(1) $$\begin{array}{cc} X & \leftarrow \mathrm{Reshape}\left(\mathrm{Concat}\left(F_{\mathrm{pre}},F_{\mathrm{post}}\right)\right),\;X\in R^{2C\times (HW)},\;F\in R^{C\times \times H\times W} \end{array}$$

(2) $$\begin{array}{cc} E & \leftarrow \mathrm{PE}(X) \end{array}$$

(3) $$\begin{array}{cc} Z & \leftarrow \mathrm{Real}({\mathrm{FFT}}_{hw}\left({\mathrm{FFT}}_{c}\left(X\right)\right)) \end{array}$$

(4) $$\begin{array}{cc} Z & \leftarrow \mathrm{Reshape}\left(\mathrm{MLP}\left(\mathrm{LN}\left(Z+E\right)\right)+X\right),\;Z\in R^{2C\times H\times W} \end{array}$$

where *F* is the image features from the encoder; *E* is the linear positional encodes from a position embedding (PE) function [10]; *Z* is the attended tensor; ${\mathrm{FFT}}_{hw}$ and ${\mathrm{FFT}}_{c}$ are the fast Fourier transform along with spatial and channel directions. Real(.) indicates the extraction of real values; LN(.) is the layer norm; and MLP(.) is the multilayer perceptron [10]. Differing from the design for natural language processing [11], the proposed PE is a linear function used to learn the positional encodes for higher dimensional features from images. In natural language processing (NLP), positional encoding (PE) is achieved using the sine and cosine function [26], as shown below:

(5) $$\begin{array}{cc} PE(pos,2i) & =sin(pos/10000^{2i/d_{model}}) \end{array}$$

(6) $$\begin{array}{cc} PE(pos,2i) & =cos(pos/10000^{2i/d_{model}}) \end{array}$$

where pos is the position and i is the dimension, according to the original article [26]. Such positional encoding is designed for sequential signals such as words and acoustic signals. The original encoding cannot satisfy the demand for higher-dimensional signals such as images and videos. The learnable PE is adopted from ViT [10] to fit higher-dimensional signals, as shown below:

(7) $$\begin{array}{c} E=W*X+b \end{array}$$

where *E* is the embedded position; *X* is the input;W,b are the weights and biases of the PE according to reference [10]. The *E* is automatically derived during the training phase. Compared with PE in NLP, the learnable PE is more scalable, which is prevalent in recent transformers in vision tasks [10,25]. Thus, we use the learnable PE in the proposed framework. After the alternating multi-head attention (MHA) with Fourier attention (FA), the occupied memory of the model is reduced from “out of memory” or OOM (more than 16 GB) to 2.8 GB with two 1024 × 1024 satellite images, as shown in Table 1. Meanwhile, Table 1 also compares the complexity of FA and MHA. The *N* is the length of feature maps that produce widths and heights. The *C* is the number of feature channels. Compared with MHA, the FA is more efficient when it comes to reducing the complexity length-wise and channel-wise. Specifically, FA makes the complexity from $N^{2}$ and $C^{2}$ reach Nlog(N) and Clog(C), which also significantly reduces the memory occupation [11].

After deriving the attended tensor from the pre- and post-disaster features, the features inside the tensor have drawn attention across both information sources. Thus, the tensor can be manipulated to assign the weights across pre- and post-disaster features in both spatial and channel directions for final feature fusion. Specifically, the fusion process can be described as:

(8) $$\begin{array}{cc} \; & \hat{Z}\leftarrow \sigma \left(Z\right),\;\hat{Z}\in \left[0,1\right] \end{array}$$

(9) $$\begin{array}{cc} \; & {\hat{Z}}_{\mathrm{pre}},{\hat{Z}}_{\mathrm{post}}\leftarrow \mathrm{Split}\left(\hat{Z}\right) \end{array}$$

(10) $$\begin{array}{cc} \; & Y\leftarrow {\hat{Z}}_{\mathrm{pre}}⊙F_{\mathrm{pre}}+{\hat{Z}}_{\mathrm{post}}⊙F_{\mathrm{post}},\;Y\in R^{C\times H\times W} \end{array}$$

where ⊙ is the element-wise production; σ(.) is the Sigmoid function used to constrain the *Z* from 0 to 1 for weight assignment; *Y* is the fused features combing weighted pre- and post-disaster features. Recalling the dense fusion structure applied in Figure 4a, the fused features can also be denoted as $Y=\{Y_{2},Y_{3},Y_{4},Y_{4}\}$ as outputs.

#### 2.1.3. Building Localization and Damage Assessment Head

In the EUBDA, the building localization head (BLH) is used to identify where the buildings are, while the damage assessment head (DAH) aims to further describe the damage level by generating masks from fused features. Both heads are developed based on the semantic feature pyramid network (SFPN), which can be described as follows:

(11) $$\begin{array}{cc} F_{\mathrm{pre}}^{\left(i\right)} & \leftarrow \mathrm{Resize}\left(\mathrm{Conv}\left(F_{\mathrm{pre}}^{\left(i\right)}\right)\right)\;i\in \left[2,3,4,5\right] \end{array}$$

(12) $$\begin{array}{cc} B & \leftarrow \sigma (\mathrm{Conv}\left(F_{\mathrm{pre}}^{\left(2\right)}+F_{\mathrm{pre}}^{\left(3\right)}+F_{\mathrm{pre}}^{\left(4\right)}+F_{\mathrm{pre}}^{\left(5\right)}\right)) \end{array}$$

where *B* is the binary mask for building segmentation; $F_{\mathrm{pre}}^{\left(i\right)}$ is the *i*-th pre-disaster features; Conv(.) is the CNN with 3×3 kernel size; Resize(.) is the interpolation function used to align the size of feature maps between stages; σ is the Sigmoid function. The SFPN is directly applied to BLH with pre-disaster features. Turning now to DAH, the head takes the fused features for a final damage assessment using the binary mask from BLH. Specifically, the DAH can be described as seen below:

(13) $$\begin{array}{cc} Y_{i} & \leftarrow \mathrm{Resize}\left(\mathrm{Conv}\left(Y_{i}\right)\right)\;i\in \left[2,3,4,5\right] \end{array}$$

(14) $$\begin{array}{cc} D & \leftarrow \mathrm{Softmax}(\mathrm{Conv}\left(\left(Y_{2}+Y_{3}+Y_{4}+Y_{5}\right)⊙P\right)) \end{array}$$

where *Y* is the fused feature; *D* is the damage mask after a Softmax function for a multi-class problem. Compared with BLH, the DAH applies the binary mask of building to suppress the features within the background areas. Thus, the DAH can better focus on estimate the damage level without disturbance from background environments, which also contributes to the model’s optimization.

#### 2.1.4. MC Dropout for Uncertainty Estimation

As mentioned, the conventional BDA models may not be reliable during inclement weather, which can cause missing casualties during post-disaster operations. In order to address the challenge, the damage assessment head (DAH) of the proposed framework provides an option to enable the Monte Carlo Dropout (MC Dropout) during inference [19]. Similar to regular dropout, the MC dropout aims to randomly remove neurons during model inference, which approximates the Monte Carlo sampling by simply calculating mean and variance after *T* times inference. In this case, the variance is treated as the uncertainty of the prediction. The process of uncertainty quantification via MC dropout during model inference can be simply defined as

(15) $$\begin{array}{cc} \; & D=\mathrm{Softmax}(\mathrm{Conv}\left(\mathrm{Dropout}\left(\sum_{i=2}^{5}Y_{i},p\right)\right)) \end{array}$$

(16) $$\begin{array}{cc} \; & \mathrm{Var}=\sum_{t=1}^{T}\frac{{\left(D_{t}-{\bar{D}}_{t}\right)}^{2}}{\left(T-1\right)},\;D∼\mathrm{Bernoulli}\left(Y\right) \end{array}$$

where *T* is the sampling times; *p* is the dropout rate; and Var is the variance or uncertainty of output D from DAH when dropout is functioning; $\bar{D}$ is the mean output. The applied random dropout enables the network to approximate variational inference between input and output. Then, the sampling process can be implemented to extract the mean and variance of the posterior distribution [19]. Finally, the variance is visualized as an uncertainty map for further processing. During the standard model inference, the dropout layer will be closed. If the robust operation is required, the dropout layer will be launched in the inference stage for the purpose.

#### 2.1.5. Objective Function

As mentioned, the proposed EUBDA consists of two predictive heads, i.e., BLH and DAH. The BLH generates a binary segmentation mask to indicate where the buildings are. On the contrary, the DAH outputs the multi-class mask to reflect the severity of the building damages. In empirical research, cross-entropy (CE) loss [1] can solve both binary and multi-class problems, which can be formulated as follows:

(17) $$CE\left(y,p\right)=-\sum_{i=1}^{N}y_{i}\log\left(p_{i}\right)$$

where *y* is the true mask; *p* is a predictive mask, and *N* is the number of classes. However, the houses are usually small blocks in the aerial images that cause the imbalance between foregrounds and backgrounds. Such imbalance may result in sub-optimal convergence during training. Thus, the dice loss (DL) [27] and focal loss (FL) [28] are introduced to address the imbalanced problems of binary classes in BLH and multiple classes in DAH. Their formulations are illustrated as below:

(18) $$\begin{array}{cc} DL(y,p) & =1-\frac{2\times \sum_{i=1}^{N}y_{i}p_{i}}{\sum_{i=1}^{N}y_{i}+\sum_{i=1}^{N}p_{i}+\epsilon } \end{array}$$

(19) $$\begin{array}{cc} FL(y,p) & =-\sum_{i=1}^{N}\alpha {\left(1-p_{i}\right)}^{\gamma }\log\left(p_{i}\right) \end{array}$$

where ϵ is the arbitrarily small number; α and γ are the hyper-parameters used to adjust the weights between hard and easy samples. These hyper-parameters are set as α=0.25 and γ=2.0 according to the original article [28], respectively. More details about these losses can be found in [27,28]. Finally, the overall loss function can be resolved as

(20) $$\begin{array}{cc} L & =L_{BLH}\left(y_{b},p_{b}\right)+L_{DAH}\left(y_{d},p_{d}\right)\\ \; & =\underset{BLH}{\underset{︸}{CE\left(y_{b},p_{b}\right)+DL\left(y_{b},p_{b}\right)}}+\underset{DAH}{\underset{︸}{CE\left(y_{d},p_{d}\right)+FL\left(y_{d},p_{d}\right)}} \end{array}$$

where $y_{b},p_{b}$ are the groundtruth and prediction of buildings; $y_{d},p_{d}$ are the groundtruth and estimation of damage.

### 2.2. Robust Operation for Supporting Final Disaster Responses

**Figure 5:**
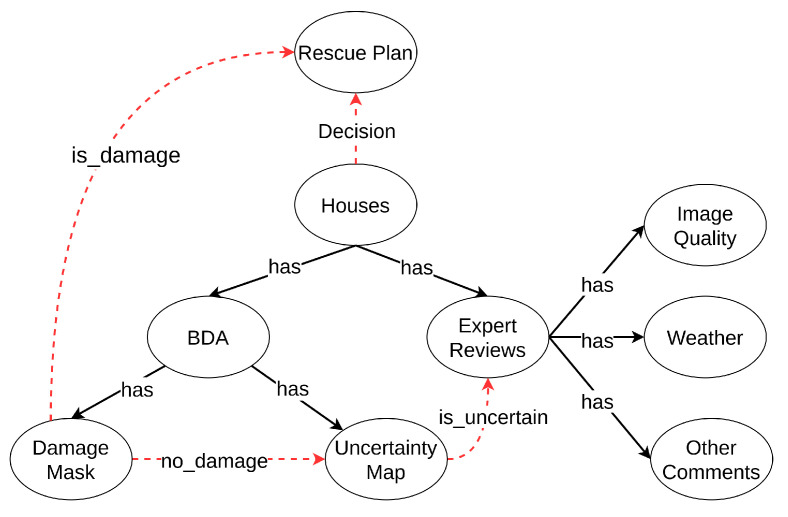
The illustration of the logical ontology in robust operation. The dashed arrow indicates the logical flow between entities.

**Figure 6:**
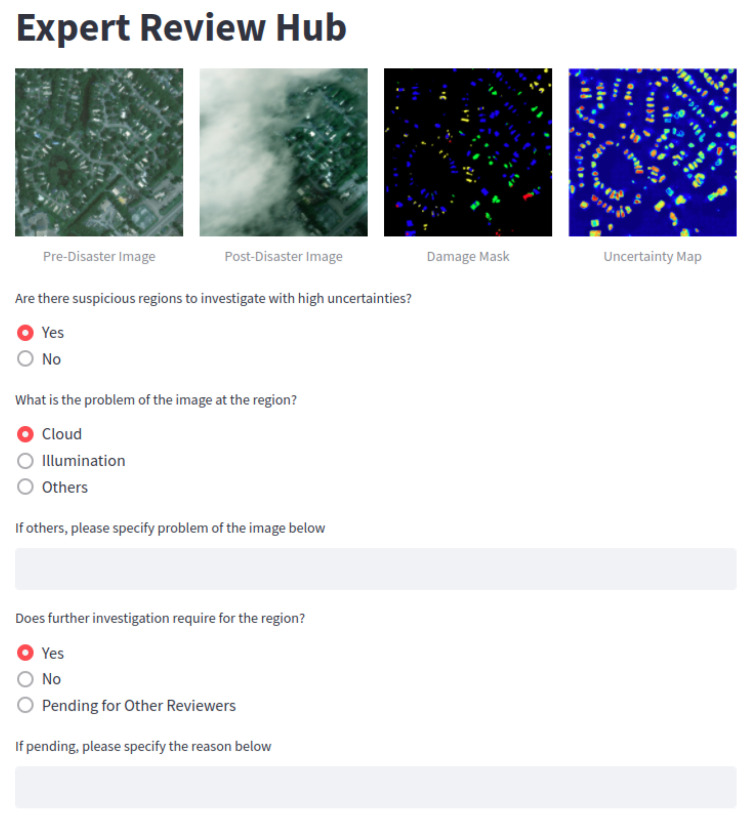
The illustration of website interface for expert reviews.

Robust operation (RO) is a way to decide if expert review is needed, according to the automatic assessment from previous EUBDA in case of false assessment. Figure 5 visualizes the structure or ontology of the process at the software level. These ellipses are the entities or classes connected based on the relation indicated by arrows. From Figure 5, the houses have two entities, i.e., building damage assessment (BDA) and expert reviews, to support the decision of whether rescue actions are needed for the houses. Recalling the proposed EUBDA, it will generate damage and an uncertainty map for inspected buildings. If there are damaged houses, the query of a rescue plan will be sent to the headquarter and rescue teams for further investigation of the houses. On the contrary, if there is no damaged house at initial screening, the corresponding uncertainty map is evaluated to determine whether the uncertainty of the houses is above the threshold (usually set as 0.5). The high uncertainty of the prediction implies the potential false prediction due to implicit factors such as sensory failures and inclement weather. Thus, the proposed RO will set a query to let experts intervene in the process for manual reviews on original pre- and post-disaster damages. The invited experts will go through the original image pairs and make comments according to the image quality, weather, and other aspects. Figure 6 shows the website interface for the expert reviews. The brief survey include several critical questions with original image pairs, damage mask, and uncertainty maps as references. The completed survey will be enclosed with automatic results from EUBDA as a profile for final decision at rescue teams.

### 2.3. Evaluation Metrics

All experimental results are evaluated via an F1 score for both building localization and building damage assessment. Specifically, the F1 score is defined as follows:

(21) $$F1=\frac{2\times \mathrm{TP}}{2\times \mathrm{TP}+\mathrm{FP}+\mathrm{FN}}$$

where TP, FP and FN are the true positive, false positive and false negative of segmentation at pixel level, respectively. Since there are four classes in the task of building damage assessment (BDA), the F1 of BDA is derived by taking the harmonic mean of the F1 over all classes. Finally, the ${F1}_{\mathrm{all}}$ [1] is included to evaluate the general performance in building localization and BDA, which is defined as follows:

(22) Overall=0.3×Loc+0.7×Dmg

where Loc and Dmg are the overall F1, F1 of building localization and F1 of BDA. The frame per second (FPS) is included to evaluate the efficiency among the proposed and comparative methods for building damage assessment. Turning now to evaluating the design of robust operation, several cases studies are conducted to determine the effectiveness of robust operation in a qualitative manner.

## 3. Experimental Results

### 3.1. Experimental Setup

In this paper, the xBD [12] dataset is used to train and validate the proposed framework. The xBD dataset contains numerous pairs of visible satellite images (size: 1024 × 1024) covering various disasters with over 800,000 polygon annotations of building locations and damage levels [1]. The damage assessment includes four levels, i.e., “no damage”, “minor”, “major”, and “destroyed”. Although the original paper indicates 9168 pre- and post-disaster image pairs, many pairs do not have corresponding annotations. Therefore, the image pairs without annotations are filtered, while 5567 pairs remain for training. For fair comparison, all BDA models are trained on a workstation configuring Nvidia Tesla V100 (16 GB RAM) at UBC Advanced Computing Center. The implemented optimizer is AdamW [29] with 0.0005 as the learning rate and four as the batch size. Meanwhile, all image pairs (1024 × 1024) are randomly cropped into smaller patches (512 × 512) to train the BDA models in an efficient manner, while the original image pairs (1024 × 1024) are used during inference. The random horizontal split is applied to improve training effectiveness as a data augmentation technique.

### 3.2. Results in Building Damage Assessment

#### 3.2.1. Comparison with Light-Weighted Backbones

Table 2 presents the comparative studies over recent efficient backbones (or primary network). Specifically, the implemented Vovnet-19 (V19) [23], ResNet-18 (R18) [7], MobileNetV2 (MV2) [30] and EfficientNet-B0 (EB0) [31] are included this comparative study. The fusion operator is fixed to addition for a fair comparison. The addition operation aims to directly sum the features of pre- and post-disaster images in a channel-wise manner, which is a conventional way to fuse the multi-modal information [25]. By observing the results in Table 2, V19 achieves the best performance among contemporary backbones over all evaluations in BDA. Meanwhile, the V19 has a similar inference time, i.e., 0.013 s, to the fastest backbone EB0. Thus, the V19 is a suitable backbone for the proposed EUBDA framework. In addition, the larger version of V19, i.e., V39, is also validated with fixed addition as fusion operation in Table 2. The experimental results show a deeper model can improve the effectiveness of the model without much sacrifice in computational efficiency. Therefore, the VovNet-series (V19 and V39) model will be selected as the implemented backbone for further experiments.

#### 3.2.2. Comparison with Fusion Operators

The previous section discusses the impacts of the backbone selection with addition as a fixed fusion operator. In this regard, turning to comparative studies in fusion operators, the backbone is initially fixed to R18, which is a standard light-weighted backbone, in this experiment. Beyond the conventional addition, three local attention methods, i.e., Gating [32], CBAM [33], and Involution [34], are included in the comparative study. A recent efficient global attention method, i.e., SRA [35], was also included. The SRA is developed according to the self-attention mechanism in reduced latent spaces, resulting in smaller memory occupation compared with the original MHA [10]. Referring to Table 1, the implemented MHA encounters OOM even on a computing node with 16 GB as GPU memories. Thus, the MHA is not included in this comparison.

**Figure 7:**
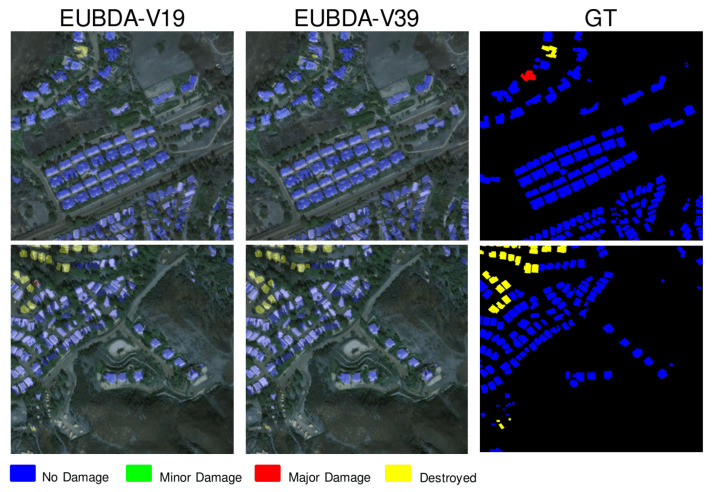
The qualitative examples of damage masks overlayed on original post-disaster images from the EUBDA with FA and VovNet (V19 and V39) as fusion operator and backbones.

Table 3 illustrates the comparative results over different fusion operators. Compared with the aforementioned state-of-the-art fusion operators, the proposed Fourier attention (FA) outperforms the rest of the contemporary attention methods. Specifically, the local attention fails to converge, resulting in poorer performance than conventional addition. On the contrary, the implemented SRA significantly improves the F1 score in building localization, damage assessment, and overall performance. Meanwhile, the SRA also achieves a balanced performance in terms of estimating four damage levels compared with the addition. The results indicate that global attention is suitable for fusing the pre- and post-disaster images. Turning now to the proposed FA, the FA outperforms the novel SRA in F1 of building localization and damage assessment. Specifically, the FA achieves around 10% improvement in overall evaluations. Although the SRA has slightly better performance, with higher F1 scores in the class of “No Damage”, the proposed FA achieves improved performance on the rest of the damage levels. In this regard, the FA has a balanced performance in BDA. Meanwhile, the FA has less inference time than SRA. Thus, the experimental results suggest that the proposed FA is the suitable fusion operator for the proposed EUBDA. Moreover, replacing R18 with V19 and deeper V39 [23] can enhance the accuracy without sacrificing inference time. Therefore, the V39 and the proposed FA are set as the backbone and fusion operator in EUBDA, respectively, for the following experiments. Some clear qualitative examples can be found in Figure 7. The qualitative results indicate that most of the areas are well segmented to classify where the damaged houses are, as shown in Figure 7. Nonetheless, there are still missing targets, as shown in the images of the first column in Figure 7. Specifically, both variants of EUBDA falsely identify the damaged house (red pixels in groundtruth) as an intact building (blue pixels in predictive masks) in Figure 7. Such false damage assessment may cause victims under ruins to be missed during the rescue operations. Thus, uncertainty quantification is still demanded to further discover the failed assessments for supporting decisions. The experimental results of uncertainty quantification are illustrated in the next section.

#### 3.2.3. Results with Additional MC Dropout

**Figure 8:**
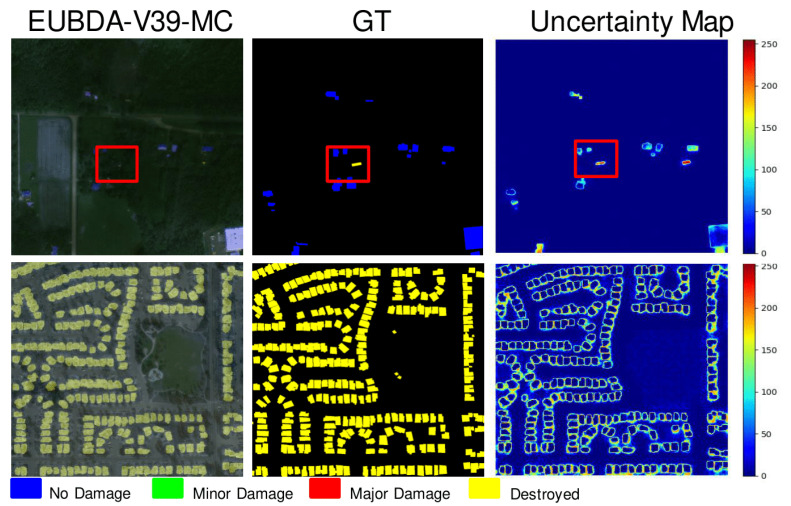
The qualitative examples of generative uncertainty maps. The houses in the red boxes indicates where the uncertainty arises due to poor illumination.

Another significant part of the EUBDA is the MC Dropout for uncertainty quantification over damage levels. The sampling rate can influence the balance between accuracy and efficiency in MC Dropout [19]. Table 4 illustrates the trade-off between accuracy and inference time when the MC Dropout is applied to our EUBDA. Compared with the vanilla approach, additional MC Dropout also facilitates the accuracy while the inference time is degraded. When the sampling rate further increases from 10 to 20, the inference time severely decreases without much improvement in F1 scores of localization and damage assessment. Thus, the sampling rate is selected as 10 for less degradation in efficiency. However, the MC Dropout enables people to interpret the results after BDA further, as shown in Figure 8. Although the house is missing after the initial BDA, the increasing uncertainty indicates the missing regions due to predictive failure in the red boxes at the first row of the Figure 8. The second row shows that the low-uncertainty regions reflect where the results are affirmed, as shown in Figure 8. More post-analysis information will be discussed in Section 3.3.

#### 3.2.4. Ablation Studies of Proposed EUBDA

Table 5 illustrates the overall ablation studies of implemented modules in EUBDA. From the observation to Table 5, the F1 scores of the segmentation are increasing with the increase in adopted modules. Especially with the proposed FA, this can greatly boost the accuracy of the EUBDA without degradation of inference time. The MC Dropout can further enhance the accuracy with additional quantified probability maps of uncertainties. Although the inference time decreases after the implementation, the predictive uncertainty enables robust operations based on the generated uncertainty maps. Therefore, the MC Dropout is dispensable from the EUBDA.

#### 3.2.5. Comparative Studies with Advanced BDA Frameworks

Through previous extensive experiments, V39 is selected as the final backbone for the proposed EUBDA, with Fourier Attention (FA) as the fusion operator. In addition, the EUBDA offers modes with and without the MC Dropout, which are included in the experiment. The variants of EUBDA are denoted as EUBDA and EUBDA-MC accordingly. The sampling rate is set as 10 to ensure a balanced performance for EUBDA-MC.

Table 6 illustrates the comprehensive comparative results with two recent advances in BDA, such as Shen et al. [1] and Weber et al. [5]. Meanwhile, three frameworks from the Xview2 challenges are also included [13,14]. Specifically, the official baseline [14], top 10 and top 1 methods in the Xview2 Challenge are also included according to published results in [14]. Although the top method is unpublished, the related source codes are uploaded in the github https://github.com/DIUx-xView/xView2_first_place (accessed on 7 September 2022). At first glance at Table 6, it is noticeable that the top method still dominates all evaluations in BDA. However, the top solution ensemble over 12 models achieves state-of-the-art results that also significantly increase the inference time. In this regard, Shen et al. [1], Weber et al. [5], and the proposed EUBDA use the end-to-end solution to accelerate the inference. Compared with solutions in Xview2 challenges, the proposed work can achieve better performance than the top 10 baseline. Turning now to the recent advanced frameworks, the proposed EUBDA has comparable performance to Weber et al. [5] with around 70% improvements in inference time. It is admitted that the proposed EUBDA still has accuracy gaps in damage assessment compared with Shen et al. [1]. However, the proposed EUBDA has distinct advantages in the application of disaster responses, as shown below:

- The proposed EUBDA can achieve similar building localization accuracy to Shen et al. [1]. Compared with Shen et al. [1], the EUBDA can achieve much faster inference speed to assist search and rescue mission coordinators in quick screening and localizing the houses within a large region.
- It is noticeable that all the frameworks are not capable of reaching full confidence in either building localization nor building damage assessment. This indicates that recent computational models still have the chance to miss buildings, which is intolerant for such a life-critical application. The proposed EUBDA offers the mode of MC Dropout (EUBDA-MC) to quantify uncertainty measurement to support decisions post-analysis, which is new to the application domain.
- Even with MC Dropout, the EUBDA-MC can still achieve faster speed than contemporary BDA frameworks.

In summary, the EUBDA regards computational models as a supplementary way to support decisions during disaster responses with faster inference and uncertainty quantification for such life-critical applications, which makes EUBDA distinguishable from the contemporary methods.

### 3.3. Case Studies in Robust Operation with Decision Support Strategy

**Figure 9:**
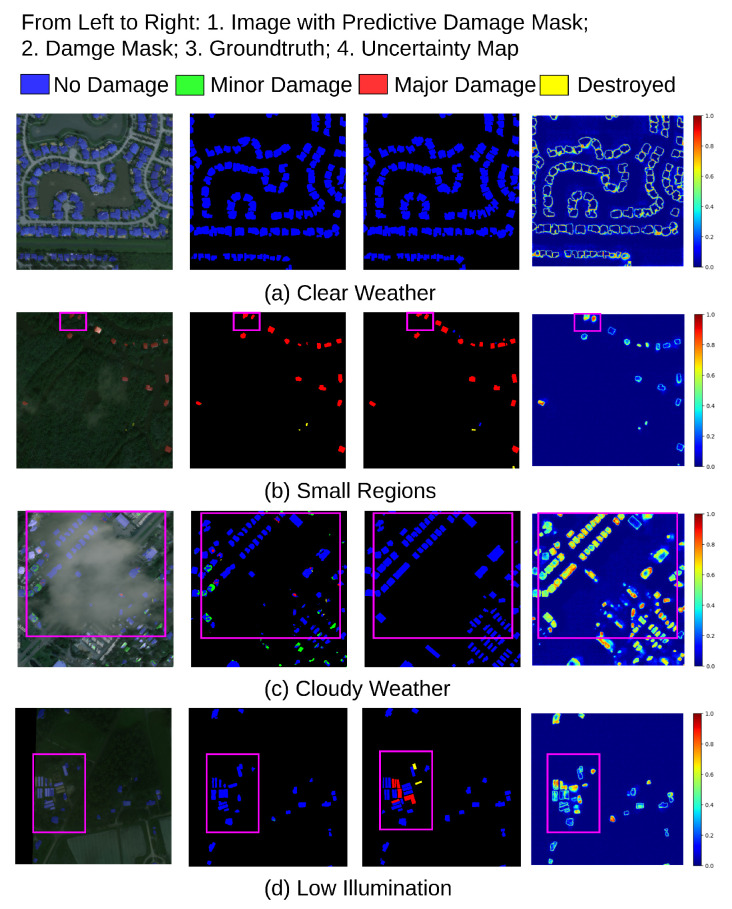
The case studies of robust operation. The pink box indicate where the experts pay attention for further investigation on the buildings.

Previous sections present how the proposed EUBDA performs in the building assessment damage (BDA) framework, which is a major part of the proposed decision support framework, namely EUDSS. With provided uncertainty maps from EUBDA, the second part of the EUDSS, i.e., robust operation (RO), aims to integrate the damage assessment mask and uncertainty maps for post-analysis, with experts recalling the Section 2.2. Figure 9 illustrates some cases for RO: (a) clear weather; (b) small regions; (c) cloudy weather; (d) low illumination. First, some buildings may not be influenced by natural or technical disasters. If the image quality is good enough between pre- and post-disaster images, the derived damaged mask can confirm that the houses are safe from the disaster. Turning to its corresponding uncertainty map, the areas of buildings that feature low values are backgrounds that indicate the confidence of this assessment is high. Thus, the region does not require an expert for further investigation, as shown in Figure 9a. However, due to the bad weather after natural disasters, the quality of post-disaster images is usually not as clear as those pre-disaster. In (b), the image is too dark to interpret where the houses are. Meanwhile, the houses are small, which causes potential missing targets. Fortunately, the EUBDA may be robust enough to achieve a satisfactory assessment compared with groundtruth. However, the uncertainty of closeness to the image border becomes significantly high in contrast with the background. With the given “Destroyed” class on the mask, the buildings inside the region are required to have a field investigation in the rescue plan. Third, Figure 9c shows that the buildings are covered by the cloud after the disaster. Although there are no damaged houses in the regions after EUBDA, the uncertainty of houses under the cloud significantly increases. Such peak uncertainty indicates the great potential of assessment failures. In this scenario, these outputs will trigger the inquiry for manual review by experts. These experts will further go through the outputs and additional information from weather stations to determine if the search and rescue plan is needed. Fortunately, the groundtruth shows there is no damaged house. Nonetheless, the RO can reduce the chance of missing damaged houses in the such life-critical applications. Figure 9d shows an example of potential target missing. The EUBDA falsely assesses that the buildings in the box are free of disaster while the houses are majorly damaged. The peak of the uncertainty triggers the expert reviews during the RO process, which can discover the faults from EUBDA. Finally, the expert makes a rescue plan for the houses to save victims from these ruins. Without the RO, the rescue teams may miss the ruins, resulting in severe consequences. In summary, the second part of the proposed EUDSS, namely robust operation (RO), greatly improves disaster response robustness empowered by uncertainty quantification.

## 4. Conclusions

This article proposes an innovative decision support system, i.e., the efficient and uncertainty-aware decision support system (EUDSS), to assist search and rescue teams in disaster responses. Specifically, the proposed EUDSS consists of BDA and robust operation (RO) stages. In the BDA stage, a computational model, namely efficient and uncertainty-aware BDA (EUBDA), is proposed to estimate damage levels and prediction uncertainties through a new Fourier attention (FA) and Monte Carlo Dropout (MC Dropout). The proposed EUBDA achieves the fastest inference speed, with competitive accuracy compared with recent advances. Moreover, unlike the contemporary methods, the results of BDA and additional uncertainty maps are revisited in the RO stage through a web application. If the results feature high uncertainty, there great potential for assessment failure. Thus, external experts’ review is required to prevent missing, damaged buildings that can cause more casualties. Several case studies are conducted to show the effectiveness of the RO for disaster responses. In conclusion, the proposed EUDSS enables the BDA to improve efficient assessment and robustness in rescue operations with measured predictive uncertainties. In future work, more investigations will be conducted on human–computer interaction techniques to enhance the efficiency and convenience of expert review.

## Figures and Tables

**Table 1:** The complexity comparison of Fourier attention (FA) and multi-head attention (MHA).

Methods	Image Size	Memory	Complexity
MHA	1024×1024	OOM (above 16 GB)	$2N^{2}C+4NC^{2}$
FA	1024×1024	2.8 GB	NClog(N)+NClog(C)

**Table 2:** Comparative studies with various backbones for building damage assessment (BDA). The fusion operator is fixed to addition. The VovNet methods achieve a better overall F1 score than other backbones, while the inference time is competitive.

Backbone	Loc ↑	Dmg ↑	Overall ↑	No Damage ↑	Minor ↑	Major ↑	Destroyed ↑	Inference Time ↓
V19 [23]	0.823	0.557	0.636	0.818	0.332	0.645	0.714	0.013
R18 [7]	0.790	0.363	0.491	0.742	0.168	0.479	0.617	0.012
MV2 [30]	0.812	0.458	0.564	0.798	0.231	0.594	0.683	**0.010**
EB0 [31]	0.831	0.089	0.312	0.748	0.025	0.535	0.623	0.025
V39 [23]	**0.836**	**0.574**	**0.646**	**0.835**	**0.431**	**0.668**	**0.732**	0.016

**Table 3:** The comparative studies of fusion operators with fixed backbone. The proposed FA achieves the best performance in overall F1 score and inference time.

Backbone	Fusion	Loc ↑	Dmg ↑	Overall ↑	No Damage ↑	Minor ↑	Major ↑	Destroyed ↑	Inference Time ↓
R18	Addition	0.790	0.363	0.491	0.742	0.168	0.479	0.617	0.013
R18	Gating [32]	0.791	0.070	0.286	0.752	0.055	0.029	0.301	0.014
R18	CBAM [33]	0.791	0.023	0.261	0.609	0.008	0.232	0.029	0.014
R18	Involution [34]	0.794	0.002	0.244	0.100	0.001	0.001	0.035	0.013
R18	SRA [35]	0.798	0.541	0.620	0.832	0.343	0.532	0.712	0.023
R18	FA (ours)	0.802	0.605	0.664	0.790	0.425	0.628	0.719	0.013
V19	FA (ours)	0.851	0.621	0.690	0.820	0.434	0.646	0.740	**0.014**
V39	FA (ours)	**0.860**	**0.678**	**0.733**	**0.855**	**0.503**	**0.696**	**0.767**	0.016

**Table 4:** The illustration of the relation between sampling rate and fusion operators when MC Dropout is applied. When the sampling rate is set as 20, the assessment performance is the best, while the inference time is doubled.

Sampling Rate	Loc ↑	Dmg ↑	Overall ↑	No Damage ↑	Minor ↑	Major ↑	Destroyed ↑	Inference Time ↓
-	0.860	0.678	0.733	0.855	0.503	0.696	0.767	**0.016**
10	**0.862**	0.687	0.740	0.869	0.506	0.705	0.784	0.032
20	0.853	**0.692**	**0.740**	**0.869**	**0.515**	**0.706**	**0.786**	0.080

**Table 5:** The ablation study of the architecture in the proposed EUBDA.

V19	V39	FA	MC	Loc ↑	Dmg ↑	Overall ↑	No Damage ↑	Minor ↑	Major ↑	Destroyed ↑	Inference Time ↓	Uncertainty
✓	-	-	-	0.823	0.557	0.636	0.818	0.332	0.645	0.714	**0.013**	-
-	✓	-	-	0.836	0.574	0.646	0.835	0.431	0.668	0.732	0.016	-
-	✓	✓	-	0.860	0.678	0.733	0.855	0.503	0.696	0.767	0.016	-
-	✓	✓	✓	**0.862**	**0.687**	**0.740**	**0.869**	**0.515**	**0.706**	**0.786**	0.080	✓

**Table 6:** The comparison with recent advanced framework in building damage assessment. The best overall F1 score is achieved by top method in Xview Challenge [14] while the proposed method (EUBDA) achieves the best inference speed.

Methods	Loc ↑	Dmg ↑	Overall ↑	No Damage ↑	Minor ↑	Major ↑	Destroyed ↑	Inference Time ↓
Official Baseline [12]	0.790	0.030	0.260	0.663	0.143	0.009	0.467	-
Top-10 Method [14]	0.852	0.680	0.732	0.880	0.475	0.713	0.807	-
Top-1 Method [14]	**0.863**	**0.788**	**0.811**	**0.923**	**0.644**	**0.785**	**0.859**	0.384
Shen et al. [1]	0.864	0.752	0.789	0.923	0.578	0.76	0.869	0.174
Weber et al. [5]	0.835	0.697	0.738	0.906	0.493	0.722	0.837	0.054
EUBDA (ours)	0.860	0.678	0.733	0.855	0.503	0.696	0.767	**0.016**
EUBDA-MC (ours)	0.862	0.687	0.740	0.869	0.506	0.705	0.784	0.032

## Data Availability

Not Applicable.
